# Description of *Heterodera microulae* sp. n. (Nematoda: Heteroderinae) from China – a new cyst nematode in the *Goettingiana* group

**DOI:** 10.21307/jofnem-2020-097

**Published:** 2020-11-24

**Authors:** Wenhao Li, Huixia Li, Chunhui Ni, Deliang Peng, Yonggang Liu, Ning Luo, Xuefen Xu

**Affiliations:** 1 College of Plant Protection, Gansu Agricultural University/Biocontrol Engineering Laboratory of Crop Diseases and Pests of Gansu Province, Lanzhou, 730070, Gansu Province, China; 2 State Key Laboratory for Biology of Plant Diseases and Insect Pests, Institute of Plant Protection, Chinese Academy of Agricultural Sciences, Beijing, 100193, China; 3 Institute of Plant Protection, Gansu Academy of Agricultural Sciences, Lanzhou, 730070, Gansu Province, China

**Keywords:** *Goettingiana* group, *Heterodera*, Morphology, New species, Phylogeny, Taxonomy

## Abstract

A new cyst-forming nematode, *Heterodera microulae* sp. n., was isolated from the roots and rhizosphere soil of *Microula sikkimensis* in China. Morphologically, the new species is characterized by lemon-shaped body with an extruded neck and obtuse vulval cone. The vulval cone of the new species appeared to be ambifenestrate without bullae and a weak underbridge. The second-stage juveniles have a longer body length with four lateral lines, strong stylets with rounded and flat stylet knobs, tail with a comparatively longer hyaline area, and a sharp terminus. The phylogenetic analyses based on ITS-rDNA, D2-D3 of 28S rDNA, and *COI* sequences revealed that the new species formed a separate clade from other *Heterodera* species in *Goettingiana* group, which further support the unique status of *H. microulae* sp. n. Therefore, it is described herein as a new species of genus *Heterodera*; additionally, the present study provided the first record of *Goettingiana* group in Gansu Province, China.

Cyst-forming nematodes are the economical pests of cultivated crops and known to be reported from all the continents (Jones et al., 2013). The genus *Heterodera* was erected by Schmidt (1871) and currently contains about 80 species (Subbotin et al., 2010). Literature studies have indicated the presence of 14 *Heterodera* species from China mainland, including *H. avenae* (Chen et al., 1991), *H. glycines* (Liu et al., 1994), *H. sinensis* (Chen and Zheng, 1994), *H. filipjevi* (Li et al., 2010), *H. koreana* (Wang et al., 2012; Wang et al., 2012b), *H. elachista* (Ding et al., 2012), *H. ripae* (Wang et al., 2012a; Wang et al., 2012b), *H. hainanensis* (Zhuo et al., 2013), *H. fengi* (Wang et al., 2013), *H. guangdongensis* (Zhuo et al., 2014), *H. zeae* (Wu et al., 2017), *H. sojae* (Zhen et al., 2018), *H. schachtii*, and *H. vallicola* (Peng et al., 2020).

Due to overlapping morphological characters and phenotypic plasticity, it is difficult to distinguish closely related *Heterodera* species; therefore, sequence-based diagnosis is gaining more reliability for precise and accurate identification of cyst-forming nematodes (Peng et al., 2003). The internal transcribed spacer region of the ribosomal DNA (ITS-rDNA), the D2 and D3 expansion fragments of the 28S ribosomal DNA genes (D2-D3 of 28S-rDNA), and mitochondrial DNA (*COI* gene) units are good candidate genes for molecular taxonomic and phylogenetic studies (Subbotin et al., 2001; Subbotin et al., 2006; Madani et al., 2004; Vovlas et al., 2017). Based on morphomolecular characterizations, Handoo and Subbotin (2018) divided *Heterodera* into nine distinct groups such as *Afenestrata*, *Avenae*, *Bifenestra*, *Cardiolata*, *Cyperi*, *Goettingiana*, *Humuli*, *Sacchari*, and *Schachtii* group. Sequence analysis of these groups is significant to study the phylogenetic relationship and identifying the *Heterodera* species.

During 2018 and 2019, a population of cyst nematode was collected from the rhizosphere of *Microula sikkimensis* in Tianzhu county of Gansu Province, China. Considering the economic value of the cyst nematode, morphomolecular studies were performed; the preliminary studies indicated that the population belongs to *Goettingiana* group of *Heterodera*. The species characters were then compared with all the related species and concluded that this population possess unique characters and it is described herein as *Heterodera microulae* sp. n.

## Materials and methods

### Isolation and morphological observation of nematodes

The nematodes were extracted from root and soil samples of *Microula sikkimensis* in Tianzhu county, Gansu Province, China. Cysts and white females were collected using sieving-decanting method, while second-stage juveniles (J2s) were recovered from hatched eggs and kept in water suspension until further use (Hooper, 1970; Golden, 1990). Males were not found. For morphometric studies, second-stage juveniles were killed by gentle heating, fixed in TAF solution (formalin: triethanolamine: water = 7:2:91), and processed to ethanol-glycerin dehydration according to Seinhorst (1959) as modified by De Grisse (1969) and mounted on permanent slides. Vulval cones were mostly mounted in glycerin jelly. Measurements were made on mounted specimens using a Nikon Eclipse E100 Microscope (Nikon, Tokyo, Japan). Light micrographs and illustrations were produced using a Zeiss Axio Scope A1 microscope (Zeiss, Jena, Germany) equipped with an AxioCam 105 color camera and Nikon YS 100 with a drawing tube (Nikon, Tokyo, Japan), respectively.

### Molecular analyses

DNA samples were prepared according to Maria et al. (2018). Three sets of primers (synthesized by Tsingke Biotech Co. Ltd., Xi’an, China) were used in the PCR analyses to amplify sequences of the ITS, D2-D3 expansion segments of 28S, and *COI* gene. The ITS region was amplified with TW81 (5′-GTTTCCGTAGGTGAACCTGC-3′) and AB28 (5′-ATATGCTTAAGTTCAGCGGGT-3′) (Maafi et al., 2003). The 28S D2-D3 region was amplified with the D2A (5′-ACAAGTACCGTGAGGGAAAGTTG-3′) and D3B (5′-TCGGAAGGAACCAGCTACTA-3′) (De Ley et al., 2005; Ye et al., 2007). Finally, the partial *COI* gene was amplified using primers Het-coxiF (5′-TAGTTGATCGTAATTTTAATGG-3′) and Het-coxiR (5′-CCTAAAACATAATGAAAATGWGC-3′) (Subbotin, 2015). PCR conditions were as described by Ye et al. (2007), De Ley et al. (2005), and Subbotin (2015). PCR products were separated on 1% agarose gels and visualized by staining with ethidium bromide. PCR products of sufficiently high quality were purified for cloning and sequencing by Tsingke Biotech Co. Ltd., Xi’an, China. The PCR products were purified by the Tiangen Gel Extraction Kit (Tiangen Biotech Co. Ltd., Beijing, China), cloned into pMD18-T vectors and transformed into DH5α-competent cells, and then sequenced by Tsingke Biotech Co. Ltd (Xi’an, China).

### Sequence alignment and phylogenetic analysis

The newly obtained sequences for each gene (ITS-rDNA, D2-D3 region of 28S-rDNA, and *COI* gene) were compared with known sequences of *Heterodera* using BLASTn homology search program. Outgroup taxa for phylogenetic analyses were selected based on the previously published studies (Subbotin et al., 2001; Maafi et al., 2003; Mundo-Ocampo et al., 2008; Kang et al., 2016; Madani et al., 2018; Vovlas et al., 2017). The selected sequences were aligned by MAFFT (Kazutaka and Standley, 2013) with default parameters and edited using Gblock (Castresana, 2000). Phylogenetic analyses were based on Bayesian inference (BI) using MrBayes 3.1.2 (Huelsenbeck and Ronquist, 2001). The GTR + I + G model was selected as the best-fit model of DNA evolution for both 28S D2-D3, ITS, and COI regions using MrModeltest version 2.3 (Nylander, 2004), according to the Akaike information criterion (AIC). BI analysis for each gene was initiated with a random starting tree and run with four Markov chains for 1,000,000 generations. The Markov chains were sampled at intervals of 100 generations and the burn-in value was 25%. Two runs were performed for each analysis. After discarding burn-in samples, the remaining samples were used to generate a 50% majority-rule consensus tree. Posterior probabilities (PP) were given on appropriate clades. The phylogenetic consensus trees were visualized using FigTree v.1.4.3 software (http://tree.bio.ed.ac.uk/software/figtree/) (Rambaut, 2016). The species in *Goettingiana* group and their localities, hosts, and GenBank accession numbers used in this study were presented in Table S1.

**Table S1.** *Goettingiana* group species, locality, host plants, and GenBank accession number used in this study.

Species	Locality	Host-plant	Marker	Accession number
*H. goettingiana*	Germany	*Pisum sp.*	ITS	AF274414
*H. goettingiana*	Lorestan, Doroud, Akbar Abad, Iran	*Trifolium repens*	ITS	AF498374
*H. goettingiana*	Monopoly, Bari province, Italy	*Pisum sativum*	ITS	KY129827
*H. sp*	Yuekou village, Tianmen county, Hubei province, China	–	ITS	HM560794
*H. sp*	Morroco	–	ITS	AY347918
*H. carotae*	Creances, France	*Daucus sp.*	ITS	AF274413
*H. carotae*	South Africa	*Daucus carota*	ITS	MG976790
*H. cruciferae*	Brielle, The Netherlands	*Brassica sp.*	ITS	AF274411
*H. urticae*	Diksmuide, Belgium	*Urtica sp.*	ITS	AF274412
*H. cruciferae*	Moscow, Russia	*Brassica oleracea*	ITS	GU126668
*H. sp*	Xinzhuang village, Huangzhong county, Qinghai province, China	–	ITS	EU623623
***H. microulae*** **sp. n.**	**Tianzhu county, Gansu province, China**	***Microula sikkimensis***	**ITS**	**MT573437**
*H. microulae* sp. n.	Haiyan county, Qinghai province, China	–	ITS	HM560791
*H. microulae* sp. n.	Haibei city, Qinghai province, China	–	ITS	HM370425
*H. microulae* sp. n.	Xining city, Qinghai province, China	–	ITS	HM370423
*H. persica*	Tehran, Dizin, Iran	*Heracleum persicum*	ITS	AF498377
*H. scutellariae*	Bremen, Germany	*Circaea lutetiana*	ITS	AY368994
*H. circeae*	Muenster, Germany	*Scutellaria galericulata*	ITS	AY368995
*H. carotae*	Ontario province, Canada	*Daucus carota*	28S	KX463292
*H. carotae*	Ontario province, Canada	*Daucus carota*	28S	KX463293
*H. sp*	Yuekou village, Tianmen county, Hubei province, China	–	28S	HM560857
*H. sp*	Iran	–	28S	GU456692
*H. cruciferae*	Iran	–	28S	KP114546
*H. urticae*	Belgium	–	28S	DQ328696
*H. goettingiana*	Iran	–	28S	DQ328697
*H. microulae* sp. n.	Haiyan county, Qinghai province, China	–	28S	HM560855
*H. microulae* sp. n.	Haomen village, Menyuan county, Qinghai province, China	–	28S	HM560856
***H. microulae*** **sp. n.**	**Tianzhu county, Gansu province, China**	***Microula sikkimensis***	**28S**	**MT573436**
*H. carotae*	South Africa	*Daucus carota*	*COI*	MN820659
*H. carotae*	Mesola, Forli-Cesena province, Italy	*Daucus carota*	*COI*	KX463299
*H. carotae*	Mesola, Forli-Cesena province, Italy	*Daucus carota*	*COI*	KX463300
*H. carotae*	Margherita, di Savoia, Italy	*Daucus carota*	*COI*	KX463301
*H. carotae*	Margherita, di Savoia, Italy	*Daucus carota*	*COI*	KX463302
*H. carotae*	Ontario province, Canada	*Daucus carota*	*COI*	KX463303
*H. carotae*	Ontario province, Canada	*Daucus carota*	*COI*	KX463304
*H. carotae*	Ontario province, Canada	*Daucus carota*	*COI*	KX463305
*H. carotae*	Ontario province, Canada	*Daucus carota*	*COI*	KX463306
*H. carotae*	Mexico	*Daucus carota*	*COI*	MG563227
*H. carotae*	Mexico	*Daucus carota*	*COI*	MG563229
*H. carotae*	Switzerland	–	*COI*	MG563231
*H. carotae*	Switzerland	–	*COI*	MG563232
*H. carotae*	France	–	*COI*	MG563233
*H. carotae*	Belgium	–	*COI*	KC172916
*H. urticae*	Faulkner county, Arkansas, USA	*Stellaria media*	*COI*	MK093155
*H. urticae*	Faulkner county, Arkansas, USA	*Stellaria media*	*COI*	MK093156
*H. cruciferae*	California, USA	–	*COI*	MG563230
*H. cruciferae*	Moscow region, Russia	–	*COI*	MG563234
*H. goettingiana*	Monopoly, Bari province, Italy	*Vicia faba*	*COI*	KY129829
*H. goettingiana*	Monopoly, Bari province, Italy	*Pisum sativum*	*COI*	KY129830
*H. goettingiana*	Monopoly, Bari province, Italy	*Medicago lupulina*	*COI*	KY129831
***H. microulae*** **sp. n.**	**Tianzhu county, Gansu province, China**	***Microula sikkimensis***	***COI***	**MT576084**

**Note:** Newly added sequences are indicated by bold font.

## Results

### Systematics

**Figure 1:**
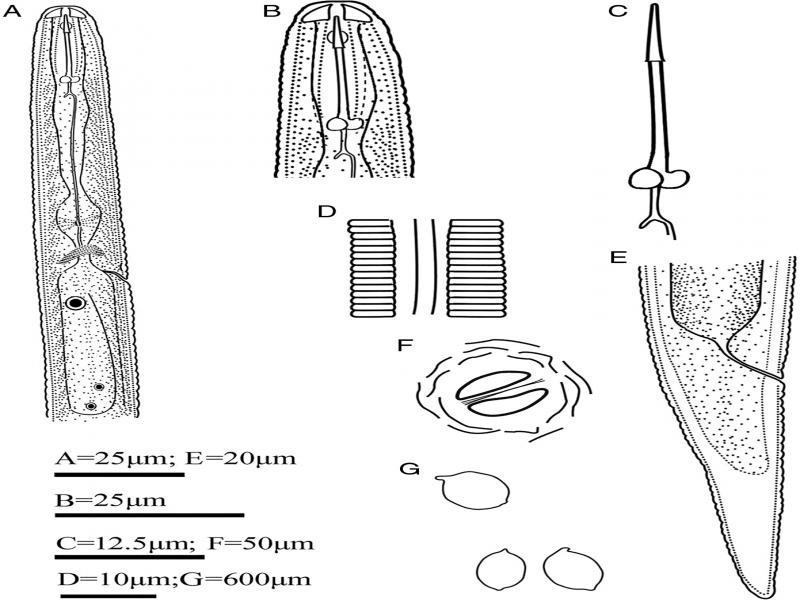
Line drawing of *H. microulae* sp. n. A: Anterior region of second-stage juvenile; B: Head of second-stage juvenile; C: Stylet of second-stage juvenile; D: Tail of second-stage juvenile; E: Cyst; F: Fenestration in vulval cone.

**Figure 2:**
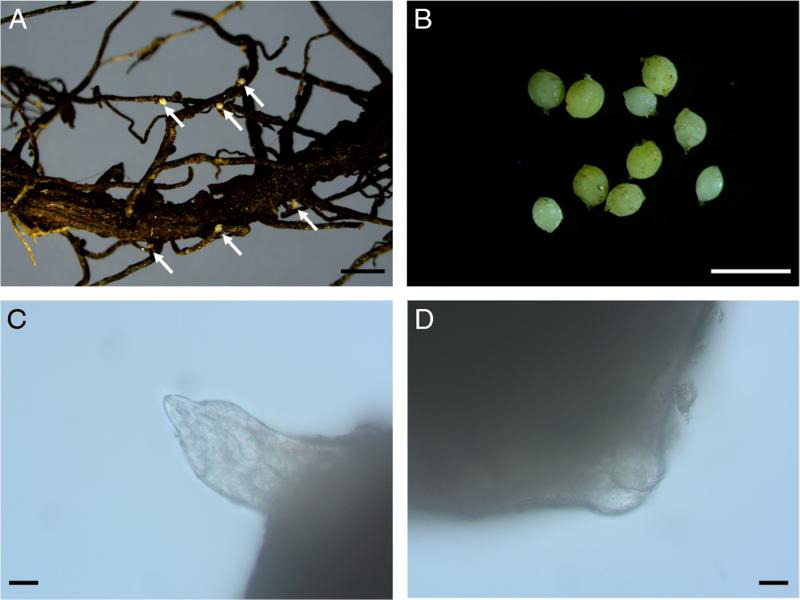
Light micrographs of *H. microulae* sp. n. A: females attached on *M. sikkimensis*; B: yellow and white females; C: Anterior region of female; D: Vulval region of female (scale bar: A = 2 mm; B = 1 mm; C, D = 20 µm).

**Figure 3:**
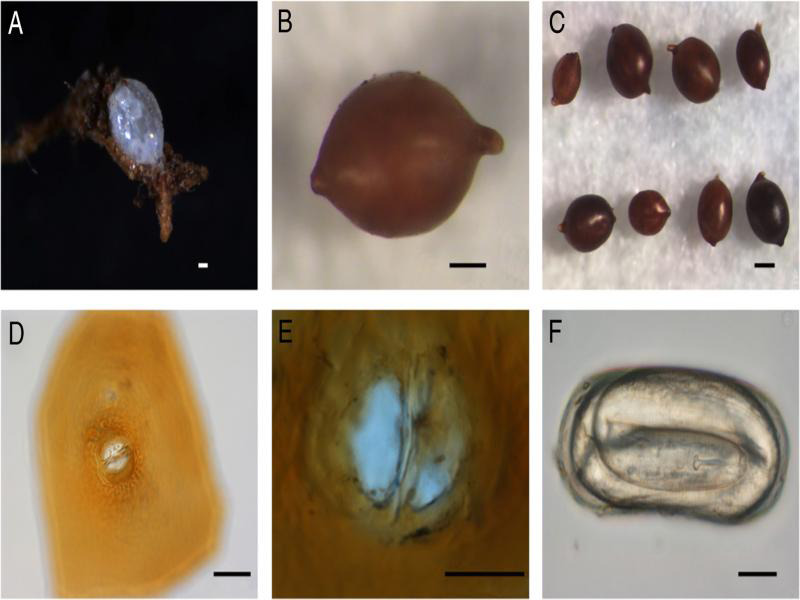
Light micrographs of *H. microulae* sp. n. A: immature female on the root; B: Cyst; C: Cysts; D-E: Fenestration in vulval cone; F: Egg (scale bar: A, D = 50 µm; B = 100 µm; C = 200 µm; E, F = 20 µm).

**Figure 4:**
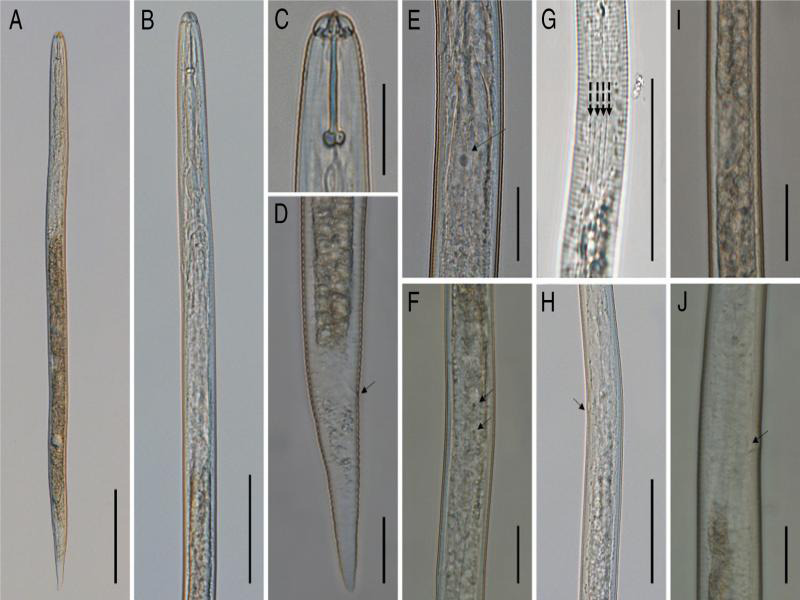
Light micrographs of second-stage juvenile of *H. microulae* sp. n. A: Entire body; B: Anterior region of; C: Head region; D: Tail region; E: Posterior pharyngeal region arrow showing the position of dorsal gland nucleus; F: Posterior pharyngeal region arrow showing the position of subventral gland nuclei; G: Lateral field; H: Hemizonid; I: Genital primordium; J: Excretory pore (scale bar: A = 100 μm, B, H, G = 50 μm, C, D, E, F, I, J = 20 μm).

*Heterodera microulae* sp. n. (Figures 1–4; Measurement Table 1)

### Description

#### Cyst

It is lemon-shaped with an obtuse vulval cone, neck extruding, and cuticle thick with an irregular zig-zag pattern. The color was white to pale to medium brown; remnants of the subcrystalline layer were rarely present. The egg sac was usually absent (Figs. 1G, 3B, C). The vulval cone was ambifenestrate-like waning crescent moon and separated by a well-developed vulval bridge. The anus area was distinct, bullae were absent (Figs. 1F, 3D, E). The vulval slit was longer than fenestral width (39.00 vs 37.75 µm); the underbridge was weak and often lost during cone preparation.

#### Female

The female was lemon-shaped, pearl white, or pale yellow in color. It was rarely rounded with a protruding neck and vulva, the subcrystalline layer was present, and the egg sac absent (Figs. 2A, B, 3A). There was a labial region with two annuli. Labial sclerotization was weak, the stylet was strong, and basal knobs were rounded and anteriorly flattened. The excretory pore was indistinct, median bulb was rounded and massive, and other parts of the pharynx were not clearly discernable. There was vulval slit in a cleft on the cone terminus (Fig. 2C, D).

#### Second-stage juvenile

The body was straight or slightly curved ventrally after heat treatment (Fig. 4A). The lip region was offset and rounded, measuring 3.90 to 5.50 (4.63) µm in height and 9.65 to 12.75 (11.01)-µm wide. The cephalic framework was strongly sclerotized (Figs. 1B, 4C). The stylet was strong; knobs were well developed, rounded and flat, or slightly concave anteriorly (Figs. 1C, 4C). The dorsal esophageal gland orifice measured from 5.32 to 6.32 (5.61) µm posterior to the stylet knob. Median bulb was rounded with a strong valvular apparatus. The pharyngeal glands were well developed, overlapping the intestine dorsoventrally (Figs. 1A, 4B). The hemizoind was distinct from one to three annuli long (Fig. 4H), the excretory pore was situated 102.46 to 130.79 (114.40) µm from the anterior end, and one to two annules were posterior to the hemizonid (Fig. 4G). There was a lateral field with four incisures (Figs. 1D, 4G). The dorsal gland nucleus and subventral gland nuclei were distinct (Fig. 4E, F). Genital primordium situated at 59 to 62% of body length behind the anterior end, with two distinct nucleate cells (Fig. 4I). The tail was conoid, gradually tapering to a finely rounded terminus. The hyaline portion was irregularly annulated occupying 50% of tail length. Phasmid was absent (Figs. 1E, 4D).

#### Eggs

Body hyaline without any markings was presented; juveniles folded six times (Fig. 3F).

#### Male

The male was not found.

#### Type material

Holotype and paratype material (20 cysts, 20 females, and 20 second-stage juveniles) were deposited in the nematode collection of the Department of Plant Protection, Biocontrol Engineering Laboratory of Crop Diseases and Pests of Gansu Province, Lanzhou, China.

#### Type host and locality

*Heterodera microulae* sp. n. was collected from the roots and rhizosphere soil of *Microula sikkimensis* Hemsl. (*Boraginaceae*, *Tubiflorae*, *Metachlamydeae*) in Tianzhu county of Gansu Province, China. The geographical position is N 37°11′46″; E 102°47′6″. This site was located in continental highland with the vegetation type of meadow grassland and the soil is composed of chernozems. The climatic parameters of the site include 450 mm of average rainfall and an approximate −2 air temperature.

#### Etymology

The species is named after the host of its isolation.

#### Diagnosis and relationships

*Heterodera microulae* sp. n. is characterized by having lemon-shaped cysts that have protruding necks and obtuse vulval cones. The cysts are 414 to 543-µm long and 305 to 456-µm wide having ambifenestrate vulval cone and bullae are absent. Females are white in color with a subcrystalline layer. Second-stage juveniles are straight or slightly curved ventrally with four incisures in the lateral field. The juveniles are 506 to 628-µm long having strong stylets with well-developed rounded stylet knobs, genital primordium situated at 59 to 62% of body length, and tail 49 to 61-µm long with a hyaline portion of 24 to 33 µm. Eggs are hyaline without any markings; juveniles inside the eggs form sixfold.

The new species belongs to the *Goettingiana* group of *Heterodera*; currently, the group contains seven valid species, viz, *Heterodera goettingiana* (Liebscher, 1892), *H. carotae* (Jones, 1950), *H. cruciferae* (Franklin, 1945), *H. circeae* (Subbotin and Turhan, 2004), *H. scutellariae* (Subbotin and Turhan, 2004), *H. urticae* (Cooper, 1955), and *H. persica* (Maafi et al., 2006).

The new species differs from *H. goettingiana* by having a shorter fenestral length (31 µm vs 35 µm), absence of bullae (vs few), weak underbridge (vs 117 µm), longer J2s body length (568 µm vs 486 µm), stylet knobs rounded and flat or slightly concave anteriorly vs smoothly rounded to slightly hook-shaped with a recurved anterior surface, longer distance of median bulb from the anterior end (MB) (86 µm vs 70 µm), shorter excretory pore distance from the anterior end (114 µm vs 158 µm), and shorter length of hyaline tail portion (29 µm vs 37 µm).

The new species is differentiated from *H. carotae* by having a bigger size of cysts (495 × 384 µm vs 408 × 309 µm), shorter vulval slit length (39 µm vs 47 µm), longer J2s body length (568 µm vs 422 µm), stylet knobs rounded and flat or slightly concave anteriorly vs concave anterior face, higher MB value (86 µm vs 66 µm), longer excretory pore distance from the anterior end (114 µm vs 99 µm), and longer tail length (57 µm vs 52 µm).

The new species differs from *H. cruciferae* by having a bigger size of cysts (495 × 384 µm vs 429 × 333 µm), slightly shorter fenestral length (31 µm vs 34 µm), shorter vulval length (39 µm vs 45 µm), longer J2s body length (568 µm vs 431 µm), higher MB value (86 µm vs 68 µm), longer excretory pore distance from the anterior end (114 µm vs 101 µm), longer tail length (57 µm vs 50 µm), and longer length of hyaline tail portion (29 µm vs 25 µm).

The new species differs from *H. persica* by a shorter fenestral length (31 µm vs 47 µm), absence of bullae (vs present), shorter vulval slit length (39 µm vs 49 µm), longer J2s body length (568 µm vs 440 µm), stylet knobs (flat or concave anteriorly vs projecting slightly anteriorly, convex posteriorly), longer stylet (26 µm vs 23 µm), higher MB value (86 µm vs 70 µm), longer excretory pore distance from the anterior end (114 µm vs 103 µm), longer tail length (57 µm vs 47 µm), and longer length of hyaline tail portion (29 µm vs 24 µm).

Compared with *H. urticae*, the new species has a smaller size of cysts (495 × 384 µm vs 492 × 435 µm), vulval cone obtrusive (vs unobtrusive) and absence of egg sac (vs presence), shorter fenestral length (31 µm vs 38 µm), shorter vulval slit length (39 µm vs 46 µm), longer J2s body length (568 µm vs 541 µm), shorter DGO (8 µm vs 5 µm), and shorter excretory pore distance from the anterior end (114 µm vs 130 µm).

The new species differs from *H. circeae* having a smaller size of cysts (495 × 384 µm vs 555 × 397 µm), a shorter fenestral length (31 µm vs 43 µm), vulval slit length (39 µm vs 48 µm), longer J2s body length (568 µm vs 434 µm), stylet knobs (rounded and slightly sloping posteriorly vs rounded and flat or slightly concave anteriorly), higher MB value (86 µm vs 70 µm), longer excretory pore distance from the anterior end (114 µm vs 101 µm), longer tail length (57 µm vs 52 µm), and longer length of hyaline tail portion (29 µm vs 26 µm).

The new species differs from *H. scutellariae*, having smaller cysts (495 × 384 µm vs 560 × 424 µm), by a shorter fenestral length (31 µm vs 35 µm), vulval slit length (39 µm vs 43 µm), longer J2s body length (568 µm vs 408 µm), higher MB value (86 µm vs 62 µm), longer excretory pore distance from the anterior end (114 µm vs 89 µm), longer tail length (57 µm vs 47 µm), and longer length of hyaline tail portion (29 µm vs 25 µm).

Additionally, comparative morphological and morphometric characters of *H. microulae* sp. n. with other valid species of *Goettingiana* group are listed in Table 2.

**Table 1.** Morphometrics of *H. microulae* sp. n.

Stage	Character	Holotype	Paratype
Cyst			
	*n*		20
	*L* (excluding length)	521.79	495.50 ± 41.01 (413.93-543.23)
	Diam.	419.33	384.29 ± 43.30 (304.96-455.51)
	*L*/Diam	1.27	1.30 ± 0.09 (1.12-1.45)
	Fenestral length	30.72	31.14 ± 1.36 (28.33-32.78)
	Fenestral width	36.16	37.75 ± 1.61 (35.46-40.07)
	Vulval slit length	35.53	39.00 ± 2.78 (35.16-44.38)
Female			
	*n*		20
	Length		454.21 ± 28.32 (381.62-496.48)
	Width		326.47 ± 31.42 (256.32-421.63)
	Length/width		1.40 ± 0.12 (1.28-1.56)
Second-stage juveniles			
	*n*		20
	Body length		567.73 ± 43.24 (505.62-627.92)
	Body width at mid-body		23.19 ± 1.31 (20.39-25.43)
	*a*		24.54 ± 1.34 (21.97-27.67)
	*b*		4.37 ± 0.33 (3.91-5.00)
	*c*		10.07 ± 1.18 (8.56-12.52)
	*c′*		4.26 ± 0.33 (3.63-4.90)
	Lip-region height		4.63 ± 0.44 (3.90-5.50)
	Lip-region diam.		11.07 ± 0.82 (9.65-12.75)
	Stylet length		25.73 ± 1.21 (24.07-28.92)
	Stylet base height		2.53 ± 0.33 (2.04-3.19)
	Stylet base width		5.30 ± 0.54 (4.39-6.09)
	Median bulb from the anterior end (MB)		85.57 ± 5.02 (76.37-95.26)
	Opening of dorsal pharyngeal gland from the stylet base (DGO)		5.13 ± 0.72 (4.03-6.41)
	Excretory pore from the anterior end (EP)		114.40 ± 6.89 (102.46-130.79)
	Median bulb width (MBW)		12.33 ± 1.49 (10.51-15.82)
	Diam. at the anus		13.23 ± 1.00 (11.24-15.26)
	Tail length		56.67 ± 3.75 (48.90-60.80)
	Hyaline portion tail		28.63 ± 1.91 (24.29-30.60)
	*L*/MB		6.65 ± 0.37 (5.76-7.53)
	TL/H		1.98 ± 0.10 (1.64-2.08)
Egg			
	*n*		20
	Length		111.61 ± 8.02 (100.21-124.65)
	Width		50.34 ± 6.71 (35.64-71.51)
	Length/width		2.26 ± 0.38 (1.43-3.26)

**Note:** All measurements are in μm, and in the form: mean ± standard (range).

**Table 2.** Main morphological character of represent species from the *Goettingiana* group (all measurements are in µm).

Species	*H. goettingiana*^*a*^	*H. carotae*^*b*^	*H. cruciferae*^*c*^	*H. persica*^*d*^	*H. urticae*^*e*^	*H. circeae*^*f*^	*H. scutellariae*^*g*^	*H. microulae* sp. n.
Host	*Pisum sativum L.*	*Daucus carota var.sativa*	*Brassica oleracea L.V.capitata*	*Heracleum persicum Desf.ex*	*Urtica dioica L.*	*Circaea lutetiana*	*Scutellaria galericulata*	*Microula sikkimensis*
Locality	Germany	England	England	Iran	Northern Ireland	Germany	Germany	China
Cyst								
Size	521 × 372	408 × 309	429 × 333	533 × 380	492 × 435	555 × 397	560 × 424	495×384
Fenestral length	35	31	34	47	38	43	35	31
Underbridge length	117	90	85	104	Weak	83	86	Weak
Bullae	Few	Absent	Absent	Present	Absent	Absent	Absent	Absent
Vulval slit length	39	47	45	49	46	48	43	39
Second-stage juvenile								
Body length	486	422	431	440	541	434	408	568
*a*	25	21	–	23	23	22	21	25
Stylet knobs	Smoothly rounded to slightly hooked shaped with recurved anterior surface	Concave anterior face	Anterior face flat to concave	Rounded or projecting slightly anterior, convex posteriorly	Slightly concave anteriorly	Rounded and slightly sloping posteriorly	Slightly convave anteriorly	Rounded and flat or slightly concave anteriorly
Stylet length	25	24	24	23	27	25	24	26
Lateral line	4	4	4	4	4	4	4	4
DGO	5	5-6	–	6	8	6	5	5
MB	70	66	68	70	–	70	62	86
Excretory pore from anterior end	158	99	101	103	130	101	89	114
Tail	60	52	50	47	58	52	47	57
Hyaline portion of tail length	37	28	25	24	29	26	25	29

**Notes:** Data from: ^a^(Stone and Course, 1974); ^b^(Mathews, 1975); ^c^(Stone and Rowe, 1976); ^d^(Mathews, 1970); ^e,f^(Subbotin and Turhan, 2004); ^g^(Maafi et al., 2006).

### Molecular characterization and phylogenetic relationships

The *H. microulae* sp. n. sequences of D2-D3 region of 28 S (734 bp), ITS (993 bp), and *COI* (415 bp) gene were obtained and submitted to the GenBank.

**Figure 5:**
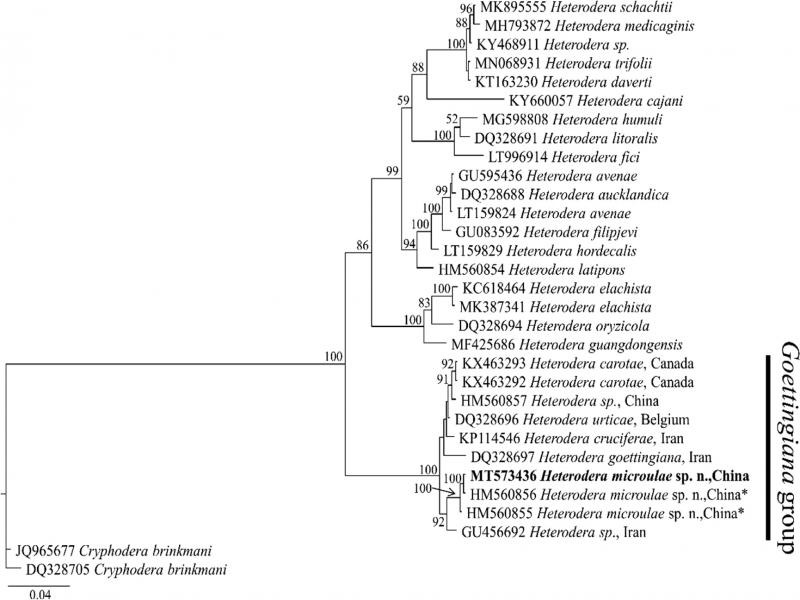
Molecular phylogenetic tree of *H. microulae* sp. n. (highlighted in bold) inferred from 28S D2/D3 extension region under GTR + I + G model. The posterior probability values exceeding 50% are given on appropriate clades. *Identified as *Heterodera sp*. by Ye et al. (unpublished) and Peng et al. (unpublished) in the GenBank.

The D2-D3 of 28S-rRNA sequence (accession no. MT573436) of *H. microulae* sp. n. showed 97.09% (19-bp difference), 97.66 to 98.49% (11-17-bp difference), 98.38% (9-bp difference), 98.62% (9-bp difference), 98.45% (11-bp difference), and 99.86 to 100% (0-1-bp difference) sequence identities with *H. goettingiana* (DQ328697), *H. carotae* (KX463292 and KX463293), *H. cruciferae* (KP114546), *H. urticae* (DQ328696), *Heterodera* sp. RH-2010 (GU456692) from Iran, and *Heterodera* sp. DP-2010 (HM560856 and HM560855) from Qinghai, China, respectively. The Bayesian phylogenetic tree of the D2-D3 of 28S gene (Fig. 5) represented a highly supported (posterior probability PP = 100) clade of *Heterodera* species, where *Goettingiana* group species occupied a basal position. It is noted that *H. microulae* sp. n. clustered together with *Heterodera* sp. DP-2010 (HM560855, HM560856) from Qinghai, China and forms a 100% supported clade.

**Figure 6:**
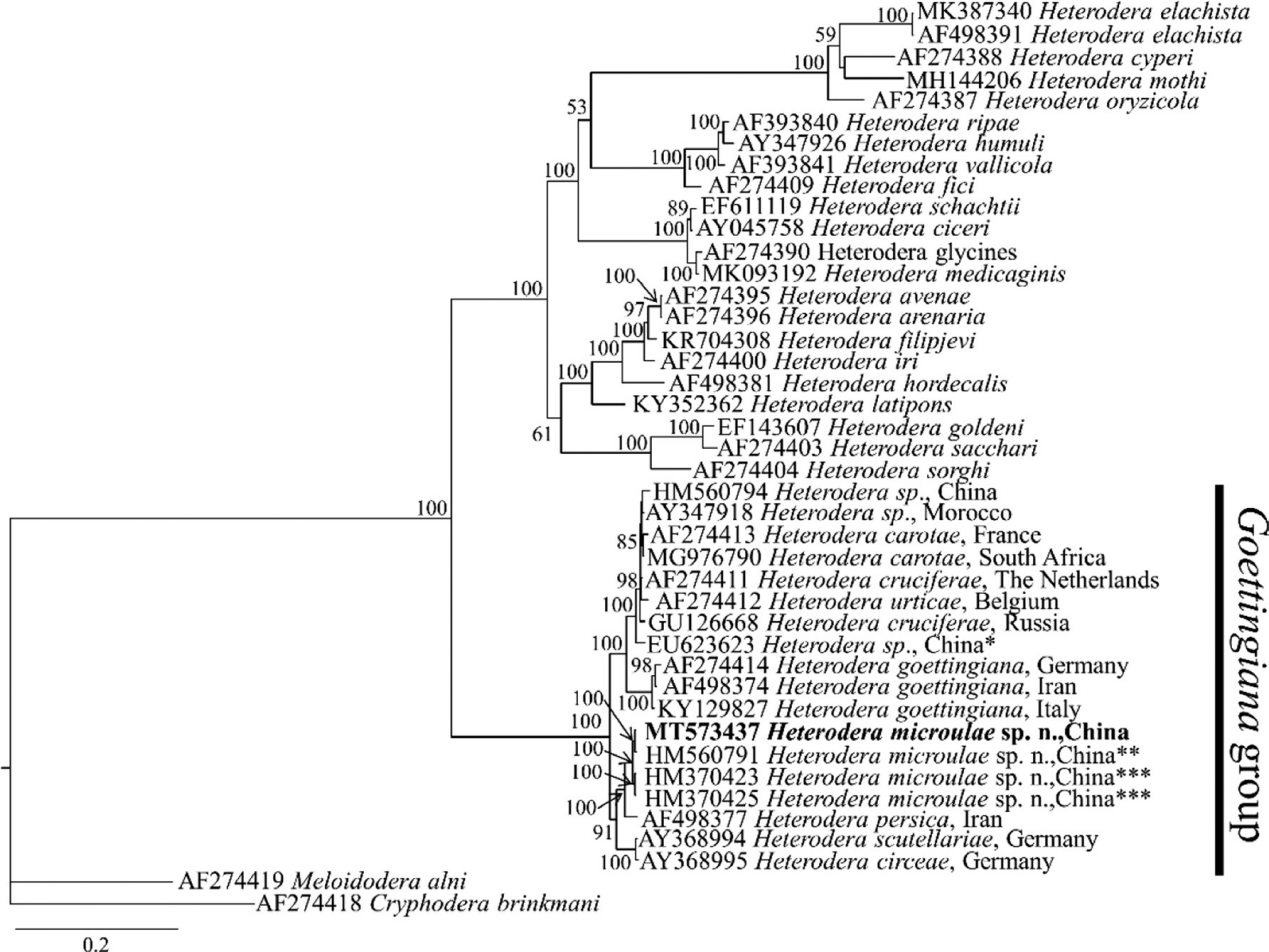
Molecular phylogenetic tree of *H. microulae* sp. n. (highlighted in bold) inferred from ITS region under GTR + I + G model. The posterior probability values exceeding 50% are given on appropriate clades. *Identified as *Heterodera goettingiana* by Peng et al. (unpublished); **Identified as *Heterodera* sp. by Peng et al. (unpublished); ***Identified as *Heterodera goettingiana* by Huang et al. (unpublished) in the GenBank.

The ITS-rDNA sequence (accession no. MT573437) divergence of *H. microulae* sp. n. with other *Goettingiana* group species is as follows: 0.20% (2-bp difference), 0.4 to 0.5% (4-bp difference), 3.02% (29-bp difference), 5.01% (48-bp difference), 5.11% (49-bp difference), 7.45% (72-bp difference), 6.77 to 6.95% (67-68-bp difference), 6.29 to 7.25% (66-70-bp difference), and 7.41 to 8% (74-77-bp difference) for *Heterodera* sp. DP-2010 (HM560761), *H. goettingiana* (HM370423, HM370425), *H. persica* (AF498377), *H. scutellariae* (AY368995), *H. circeae* (AY368994), *H. urticae* (AF274412), *H. carotae* (AF274413; MG976790), *H. cruciferae* (AF274411; GU126668), and *H. goettingiana* (KY129827; AF274411; AF498374), respectively. The Bayesian phylogenetic tree of the ITS gene (Fig. 6) represented a highly supported (posterior probability PP = 100) clade of *Heterodera* species. As in the 28S tree, the ITS tree also positioned the *Goettingiana* group species. *H. microulae* sp. n. (MT573437) clustered with *H. persica* (AF498377), *H. scutellariae* (AY368994), *H. circeae* (AY368995), *Heterodera* sp. DP-2010 (HM560791), and *H. goettingiana* (HM370423, HM370425) from Qinghai, China with high-probability support (pp = 91%). It is also noted that sequences of *H. goettingiana* (HM370423, HM370425) from Qinghai, China, clustered outside with other *H. goettingiana* (KY129827, AF274411, and AF498374) subclades and should be considered a misidentification. However, *H. microulae* sp. n. (MT573437) is clustered with *H.* sp. DP-2010 (HM560791) and *H. goettingiana* (HM370423, HM370425) from Qinghai, China, with 100% support. It is also noted that *H. microulae* sp. n. (MT573437) clustered with two Chinese populations of *Heterodera* species (HM560791; HM370425) with 100% support.

**Figure 7:**
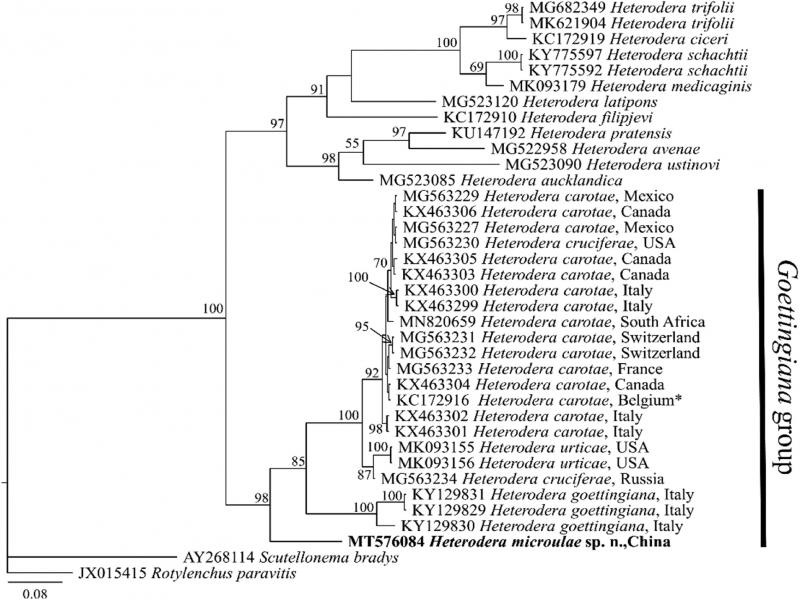
Molecular phylogenetic tree of *H. microulae* sp. n. (highlighted in bold) inferred from *COI* gene under GTR + I + G model. The posterior probability values exceeding 50% are given on appropriate clades. *KC172916 identified as *H. pratensis* by Toumi et al. (2013) and later corrected to *Heterodera carotae* by Madani et al. (2018).

The *COI* gene sequence of *H. microulae* sp. n. showed 87.21 to 89.53% (differing from 36 to 44 bp), 88.19% (differing from 43 bp), 88.67 to 88.92% (differing from 46 to 47 bp), and 88.67 to 89.40% (differing from 44 to 47 bp), sequence identities with *H. goettingiana* (KY129829-KY129831), *H. urticae* (MK093155 and MK093156), *H. cruciferae* (MG563230 and MG563234), and *H. carotae* (KX463299-KX463306, MG563227, MG563229, MG563231-MG563233, and MN820659), respectively. The Bayesian phylogenetic tree of the *COI* gene (Fig. 7) represented a highly supported (posterior probability PP = 100) clade of *Heterodera* species. In this tree, *H. microulae* sp. n. clustered with *H. goettingiana*, *H. urticae*, *H. cruciferae*, and *H. caratae* with 98% support; however, *H. microulae* sp. n. formed a separate clade from those sequences.

## Discussion

Taxonomy of *Heterodera* species has been revised extensively in the past; Baldwin and Mundo-Ocampo (1991) placed 23 *Heterodera* species into *Goettingiana* group. However, Sturhan (1998) and Subbotin et al. (2001) used J2’s lateral field characters and host preferences to separate *Heterodera* species into different groups (such as *Bifenestra*, *Cyperi*, and *Humuli* groups). The key morphological characters of the *Goettingiana* group include lemon-shaped cysts having a protruding neck, ambifenestration, and absence of bullae (small bullae occasionally present); some species may have an egg sac, vulval slit length > 35 µm, a thin vulval bridge, fenestral length (30-45 µm), and a weak underbridge. There were second-stage juveniles with body length > 400 µm, stylet length > 20 µm, tail length > 45 µm, hyaline tail portion > 20 µm, and lateral field with four lines (Subbotin and Turhan, 2004). The new species also belong to the *Goettingiana* group and morphologically very close to *H. urticae*; however, morphometrics of J2s body lengths, DGO and excretory pore position, fenestral length, vulval slit length, and cyst width can be used to differentiate both species.

Phylogenetically, it is evident that *H. microulae* sp. n. is a member of *Goettingiana* group. In our analyses, it is also noted that *Heterodera* sp. DP-2010 (HM560791, HM560855, and HM560856) and *H. goettingiana* (HM370423 and HM370425) from Qinghai, China, formed a well-supported molecular clade with the *H. microulae* sp. n. Moreover, the nucleotide differences of these sequences with our new species sequences are also very low (2-4-bp difference for ITS and 0-1 bp for 28S). Previously, Escobar-Avila et al. (2018) indicated that the sequences of *H. goettingiana* (HM370423 and HM370425) from Qinghai, China, might be a case of misidentification. Based on our phylogenetic and sequence analysis results, we regard *Heterodera* sp. DP-2010 (HM560791, HM560855, and HM560856) and *H. goettingiana* (HM370423 and HM370425) as *H. microulae* sp. n.

*Heterodera microulae* sp. n. is isolated from *Microula sikkimensis*, it is a biennial herbaceous plant that grows in forests, meadows, and forest edges at altitudes of 2,200 to 4,700 m, and it is widely distributed in South and East Asian countries (Pi et al., 2014). *H. microulae* sp. n. was found in Gansu and Qinghai Provinces, but we speculate that it is likely to be found in some localities that are characterized by low temperature, high rainfall, and high altitude.

The present study described a new species found in the rhizosphere of *M. sikkimensis*; further research is needed to understand the distribution and biology of the new species. In addition, plenty of leguminous crops (pea, kidney bean, pole bean, etc.) are growing in the same locality. Therefore, host-suitability tests of *H. microulae* sp. n. are an open research field to investigate the damage potential of this species.
